# Comparison of four diagnostic indices for metabolic syndrome in a Northern Spanish population

**DOI:** 10.1371/journal.pone.0339369

**Published:** 2026-01-09

**Authors:** Liliana Bilbie Lupchian, Bárbara Oliván-Blázquez, Edgar Peña-Galo, Marta Domínguez-García, María Antonia Sánchez-Calavera

**Affiliations:** 1 IIS Aragón (Instituto de Investigación Sanitaria Aragón - Institute for Health Research Aragón), Zaragoza, Spain; 2 Specialist in Family and Community Medicine, Aragonese Health Service, Zaragoza, Spain; 3 Department of Psychology and Sociology, University of Zaragoza, Zaragoza, Spain; 4 Research Network of Chronicity, Primary Care and Health Promotion (RICAPPS) RD21/0016/0001, Zaragoza, Spain; 5 Department of Medicine, Psychiatry and Dermatology, Faculty of Medicine, University of Zaragoza, Zaragoza, Spain; Federal University of Rio Grande do Sul: Universidade Federal do Rio Grande do Sul, BRAZIL

## Abstract

Cardiometabolic diseases are the leading cause of death worldwide, and early identification of individuals at risk is essential to reduce their burden. This study compared the performance of four diagnostic indices — the Visceral Adiposity Index (VAI), Body Mass Index (BMI), Waist Circumference (WC), and Atherogenic Index (AI) — in predicting Metabolic Syndrome (MetS) in a primary care population from northern Spain. A retrospective observational study was conducted using data from the CRONAP cohort (n = 3,107). Anthropometric and analytical parameters were analyzed using logistic regression and ROC curves. The VAI showed the highest predictive capacity for MetS (AUC = 0.779), followed by WC (0.756), BMI (0.730), and AI (0.665). In the MetS group, higher and statistically significant values were observed for glucose, glycated hemoglobin, triglycerides, blood pressure, BMI, and WC (p < 0.001), while HDL-cholesterol and glomerular filtration rate were lower. VAI outperformed the other indices in identifying MetS and may serve as a useful screening tool in primary care settings; however, as this is a retrospective, single-region study, further research is required to validate its diagnostic cut-off and generalizability to other populations.

## Introduction

Cardiometabolic diseases are the principal causes of global mortality and pose a significant economic burden on healthcare systems [1]. In Spain, Primary Care plays a fundamental role in providing comprehensive, multidisciplinary, and longitudinal care aimed at increasing both life expectancy and quality of life In this context, a key strategy to improve health outcomes is addressing avoidable morbidity. Thus, specific preventive interventions are directed toward people at high cardiometabolic risk, among whom metabolic syndrome (MetS) is a frequent and sometimes underdiagnosed condition.

European guidelines have recently emphasized the crucial role of primary care in the prevention and management of obesity and its associated comorbidities, defining obesity as a complex, multifactorial, and chronic disease that requires individualized, non-stigmatizing, and patient-centered approaches [2]. These recommendations highlight that even modest reductions in body weight or waist circumference can lead to significant metabolic improvements and reinforce the importance of using reliable anthropometric and biochemical indices—such as waist circumference or composite indicators like the Visceral Adiposity.

In recent years, metabolic syndrome (MetS) has gained renewed attention, particularly after the COVID-19 pandemic, which highlighted the strong connection between metabolic and psychological health [3]. The global health crisis led to increased sedentary behavior, dietary changes, and higher stress levels, factors that may exacerbate metabolic dysregulation. MetS represents a cluster of metabolic abnormalities—central obesity, insulin resistance, hypertension, and dyslipidemia—that significantly increase the risk of type 2 diabetes and atherosclerotic cardiovascular disease. Current epidemiological data indicate that more than one-fifth of adults in Europe and the United States are affected, reflecting the global rise in obesity and sedentary lifestyles [4]. Beyond its metabolic implications, MetS entails a substantial economic and social burden, reinforcing the urgent need for early detection and preventive interventions in primary care. In parallel, public interest in alternative or “natural” remedies has increased, sometimes leading to unsupervised practices lacking scientific evidence and posing potential health risks [5]. Identifying individuals at risk through validated and accessible diagnostic indices can help mitigate these metabolic and cardiovascular consequences.

The MetS — whose incidence continues to rise — includes insulin resistance, abdominal obesity, dyslipidemia, and arterial hypertension, and is also associated with endothelial dysfunction and atherosclerosis. These alterations increase cardiometabolic risk and promote the development of cardiovascular disease and type 2 diabetes mellitus [6,7]. Several definitions for MetS have been proposed, but the most widely used criteria are those of the International Diabetes Federation (IDF) and the National Cholesterol Education Program Adult Treatment Panel III (NCEP-ATP III) [8]. The close relationship between MetS and obesity has been extensively documented, since adipose tissue is now recognized as an active endocrine organ that secretes adipokines involved in inflammation and insulin resistance [9]. The accumulation of visceral fat—particularly around internal organs—has greater cardiometabolic consequences than subcutaneous fat, due to its higher proinflammatory and insulin-desensitizing activity [10–14]. Consequently, detecting and quantifying visceral adiposity is essential for accurate risk assessment, yet the gold-standard imaging methods are costly and not feasible in routine clinical practice [15].

Over time, several anthropometric indices have been proposed to quantify visceral fat in a simple and non-invasive way, including body mass index (BMI), waist circumference (WC), and the waist-to-height ratio (WHtR). Although BMI is the most widely used measure, several studies have questioned its accuracy in identifying individuals at cardiometabolic risk. In a large meta-analysis including more than 88,000 participants, indices of central adiposity—particularly WHtR—were shown to outperform BMI in discriminating hypertension, diabetes, and dyslipidemia [16]. These findings emphasized the importance of fat distribution rather than overall body mass. Subsequently, new composite indices incorporating both anthropometric and biochemical variables have been developed to improve the estimation of visceral adiposity and metabolic risk [4]. Among these, the Visceral Adiposity Index (VAI) stands out for combining BMI, WC, triglycerides, and HDL-cholesterol in a sex-specific formula [17]. The VAI has demonstrated strong associations with insulin resistance, type 2 diabetes, and cardiovascular disease across various populations [18–20]. Ethnic and regional differences, however, can substantially influence its predictive performance, reinforcing the need for population-specific validation [21,22]. Recent evidence from Mediterranean cohorts supports its usefulness as a practical and accessible screening tool in primary care [22].

In view of the above, further research is needed to confirm the predictive value of the Visceral Adiposity Index (VAI), an easy-to-measure, inexpensive, and non-invasive indicator of visceral fat function. Evidence in European populations remains limited, and regional variations in body composition and lipid profiles may affect diagnostic accuracy.

Therefore, the present study aimed to evaluate the predictive value of the VAI for metabolic syndrome (MetS) in a Mediterranean population, comparing it with other commonly used indices—body mass index (BMI), waist circumference (WC), and atherogenic index (AI)—to assess its clinical applicability in primary care settings.

## Methods

The data for this study were obtained from the **BIGAN (Big Data Sanitario de Aragón)** platform, a regional infrastructure managed by the **Institute of Aragonese Health Sciences (IACS)** that integrates anonymized health information from the public healthcare system of Aragón (Spain) [23]. BIGAN allows the creation of specific research cohorts derived from electronic health records (EHRs) and ensures compliance with data protection regulations under the *Orden SAN/1355/2018*. From this platform, we extracted the **CRONAP database**, which compiles information from all primary care EHRs of residents aged ≥16 years with active clinical records in the Aragón Health Service. At the beginning of the study, the CRONAP cohort included **732,585 patients**, representing nearly the entire adult population of Aragón. The database contains detailed sociodemographic, clinical, and laboratory data for individuals diagnosed with at least one chronic disease with a prevalence greater than 5%, such as hypertension, dyslipidemia, obesity, diabetes, ischemic heart disease, chronic kidney disease, or respiratory, thyroid, and mental health disorders. Importantly, patients with confirmed SARS-CoV-2 infection during the first year of the COVID-19 pandemic were excluded to analyze the evolution of chronic conditions unrelated to the infection.

To ensure methodological consistency, all individuals lacking the necessary anthropometric or biochemical variables to compute the Visceral Adiposity Index (VAI) as of September 14, 2019 — six months before the COVID-19 lockdown — were excluded from the dataset. From the CRONAP cohort, a total of 3,107 individuals with complete anthropometric and biochemical data required for VAI calculation were selected for analysis. Given the universal coverage of Spain’s healthcare system, the CRONAP database provides a highly representative sample of the adult population of Aragón and constitutes a reliable source for population-based metabolic research.

**Fig 1:**
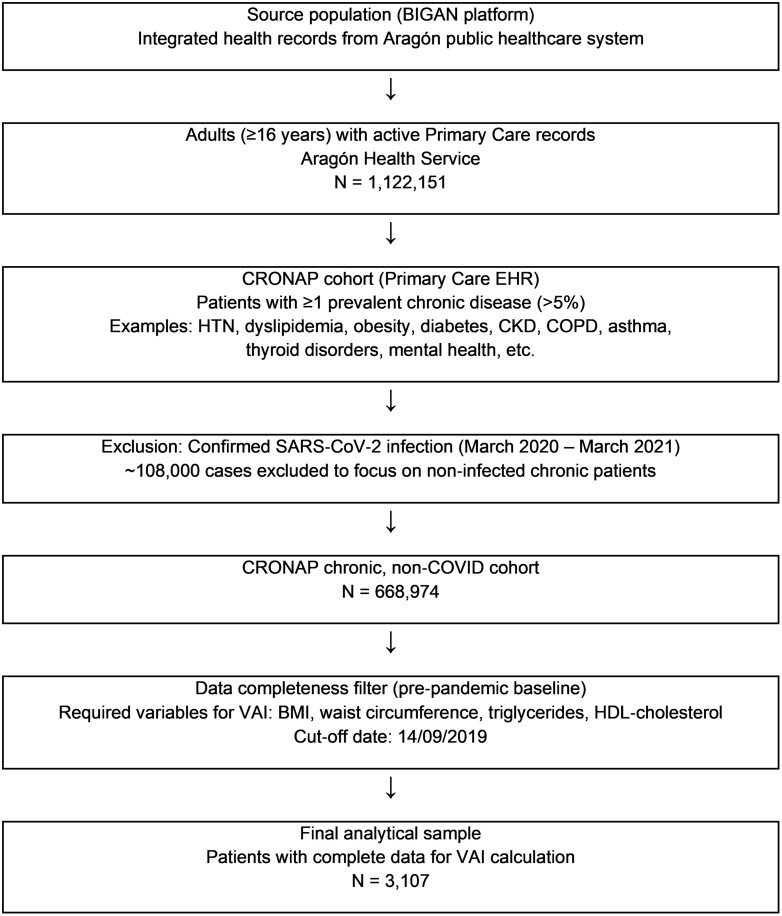
Flowchart of patient selection. Flowchart illustrating the patient selection process from the BIGAN platform and the CRONAP cohort to the final analytical sample of 3,107 individuals included in the study. Boxes represent inclusion and exclusion criteria, and arrows indicate the flow of selection.

The process of patient selection is summarized in Fig 1.

### Statistical analysis

Statistical analyses were conducted using *IBM SPSS Statistics*, version 22.0 (IBM Corp., Armonk, NY, USA). The level of statistical significance was set at *p* < 0.05, and all tests were two-tailed.

Descriptive analyses were first performed for all study variables. Categorical variables were summarized as absolute and relative frequencies, while continuous variables were expressed as means ± standard deviations (SD). Given the large sample size, parametric methods were applied, as the sampling distribution of the mean tends to approximate normality under the Central Limit Theorem, even if the raw data are not normally distributed.

A bivariate analysis was then carried out to compare participants with and without metabolic syndrome (MetS), stratified by sex. Differences between groups were assessed using the chi-square (χ²) test for categorical variables and the Student’s *t*-test for independent samples for continuous variables.

To explore independent associations between MetS and the evaluated indices—Visceral Adiposity Index (VAI), Body Mass Index (BMI), Waist Circumference (WC), and Atherogenic Index (AI)—a multivariate logistic regression model was fitted. The dependent variable was the presence of MetS, and regression coefficients were expressed as odds ratios (ORs) with corresponding 95% confidence intervals (CIs).

The models were adjusted for potential confounders identified in the literature as relevant to metabolic and cardiovascular risk. Specifically, the following covariates were included: age, sex, and rurality (sociodemographic factors), together with hypertension, diabetes mellitus, dyslipidemia, obesity, and smoking status (clinical factors). These variables were chosen based on their biological plausibility, statistical relevance, and availability in the CRONAP dataset.

The predictive performance of the four indices for identifying MetS was evaluated using receiver operating characteristic (ROC) curve analysis, and the area under the curve (AUC) was calculated with 95% CIs. The Youden index (J = sensitivity + specificity – 1) was applied to determine the optimal cut-off value of the VAI. Sensitivity, specificity, positive predictive value (PPV), and negative predictive value (NPV) were computed for this threshold.

Finally, Pearson’s correlation coefficients (r) were used to assess linear relationships between the VAI and other metabolic and anthropometric variables, providing additional insight into the strength and direction of associations within the study population.

### Definition of metabolic syndrome and indices

Metabolic syndrome (MetS) was defined according to the criteria established by the National Cholesterol Education Program Adult Treatment Panel III (NCEP-ATP III) [8].

The body mass index (BMI) was calculated as weight (kg) divided by height squared (m^2^) and categorized as < 25.0 kg/m^2^ (normal weight), 25.0–29.9 kg/m^2^ (overweight), and ≥ 30.0 kg/m^2^ (obesity).

The atherogenic index (AI) was computed as the ratio of total cholesterol to HDL-cholesterol (AI = total cholesterol/ HDL-cholesterol).

The Visceral Adiposity Index (VAI) was calculated according to the sex-specific equations proposed by Amato et al. (2010) [24]:

- For women: VAI = [WC/ (39.68 + 1.89 × BMI)] × (TG/ 1.03) × (1.31/ HDL);
- For men: VAI = [WC/ (36.58 + 1.89 × BMI)] × (TG/ 0.81) × (1.52/ HDL).

All biochemical variables were expressed in mg/dL, and waist circumference (WC) was measured in centimeters.

### Ethical approval and data protection

This study was conducted in accordance with the ethical standards of the Declaration of Helsinki and the applicable national regulations on data protection. The research protocol was reviewed and approved by the Ethics Committee for Clinical Research of Aragón (CEICA) (reference: PI21/288). The data used were obtained from the BIGAN (Big Data Sanitario de Aragón) platform, managed by the Institute of Aragonese Health Sciences (IACS), which provides anonymized and pseudonymized health information for research purposes under the Orden SAN/1355/2018. Because all data were fully anonymized before analysis, individual informed consent was not required. The study complies with the provisions of the General Data Protection Regulation (EU) 2016/679 (GDPR) and Spanish data protection laws.

## Results

From the CRONAP cohort — which includes 732,585 patients — 3,107 patients who had collected the variables necessary for the calculation of the VAI were selected. The diagnosis of MetS was established according to the NCEP-ATP III criteria, resulting in 1,624 patients (52.26% of the total) meeting them. Of these, 47.0% were women and 53% were men, with a mean age of 64.84 years (SD: 14.47). The sociodemographic data and comorbidities of the cohort, according to the presence or absence of MetS, are collected in Table 1.

**Table 1:** Comparison between patients with and without Metabolic Syndrome (MetS).

Variable	Category	MetS: Yes (n = 1624)	MetS: No (n = 1483)	p-value
**Total**		1624 (52.3%)	1483 (47.7%)	—
**Sex**	Women	764 (51.4%)	722 (48.6%)	0.360
	Men	860 (53.1%)	761 (46.9%)	
**Age (years)**	<45	79 (28.8%)	195 (71.2%)	0.006
	45–65	625 (53.5%)	544 (46.5%)	0.015
	65–85	801 (56.1%)	627 (43.9%)	0.400
	>85	119 (50.4%)	117 (49.6%)	0.012
**Living area**	Rural	733 (51.5%)	689 (48.5%)	0.459
	Urban	891 (52.9%)	794 (47.1%)	
**Comorbidities**	Heart failure	61 (61.0%)	39 (39.0%)	0.076
	Ischemic heart disease	104 (53.6%)	90 (46.4%)	0.700
	Hypertension	1084 (61.4%)	682 (38.6%)	<0.001
	Dyslipidemia	1009 (55.3%)	816 (44.7%)	<0.001
	Obesity	497 (69.3%)	220 (30.7%)	<0.001
	Overweight	29 (39.2%)	45 (60.8%)	0.023
	Cerebrovascular disease	79 (53.4%)	69 (46.6%)	0.782
	Diabetes mellitus	744 (72.3%)	285 (27.7%)	<0.001
	Chronic kidney disease	154 (53.8%)	132 (46.2%)	0.575
	Smoking	256 (48.3%)	274 (51.7%)	0.045
	Alcoholism	27 (62.8%)	16 (37.2%)	0.164

**Legend:** Data are expressed as number (percentage). Comparisons between groups were performed using the chi-square (χ²) test for categorical variables and Student’s t-test for independent samples for continuous variables. Statistically significant values (p < 0.05) are shown in bold.

**Abbreviations:** MetS, metabolic syndrome; BMI, body mass index; WC, waist circumference; HDL, high-density lipoprotein cholesterol; LDL, low-density lipoprotein cholesterol; SBP, systolic blood pressure; DBP, diastolic blood pressure; GFR, glomerular filtration rate; AI, atherogenic index; VAI, visceral adiposity index.

Total cholesterol was 195.8 ± 44.5 mg/dL in patients with MetS and 198.6 ± 40.8 mg/dL in those without MetS (p = 0.073; 95% CI: –5.80 to 2.58).

HDL-cholesterol was 49.8 ± 13.1 mg/dL in the MetS group and 58.4 ± 17.5 mg/dL in the non-MetS group (p < 0.001; 95% CI: 7.49 to 9.65).

LDL-cholesterol was 115.4 ± 37.9 mg/dL in the MetS group and 120.3 ± 35.1 mg/dL in the non-MetS group (p < 0.001; 95% CI: 2.28 to 7.52).

Fasting glucose, glycated hemoglobin (HbA1c), triglycerides, systolic blood pressure (SBP), and diastolic blood pressure (DBP) were higher in the MetS group, whereas the glomerular filtration rate (GFR) was lower. All differences were statistically significant (p < 0.001). The corresponding values and 95% confidence intervals are presented in Table 2.

**Table 2:** Comparison of clinical and biochemical variables according to the presence of Metabolic Syndrome (MetS).

Variable	All cases (mean ± SD)	MetS: Yes (n = 1624)	MetS: No (n = 1483)	p-value	95% CI (Lower)	95% CI (Upper)
**HDL-cholesterol (mg/dL)**	53.9 ± 15.9	49.8 ± 13.1	58.4 ± 17.5	<0.001	7.49	9.65
**LDL-cholesterol (mg/dL)**	117.7 ± 36.7	115.4 ± 37.9	120.3 ± 35.1	<0.001	2.28	7.52
**Total cholesterol (mg/dL)**	197.1 ± 42.8	195.8 ± 44.5	198.6 ± 40.8	0.073	−0.26	5.80
**Fasting glucose (mg/dL)**	111.8 ± 33.7	123.4 ± 36.2	99.2 ± 25.3	<0.001	−26.43	−21.96
**Glycated hemoglobin (HbA1c, %)**	6.8 ± 1.2	7.02 ± 1.2	6.4 ± 1.1	<0.001	−0.66	−0.36
**Triglycerides (mg/dL)**	130.8 ± 74.4	157.2 ± 83.5	102.1 ± 48.8	<0.001	−60.02	−50.28
**Glomerular filtration rate (mL/min/1.73m²)**	81.7 ± 19.7	80.4 ± 20.1	83.1 ± 19.0	<0.001	1.28	4.10
**Systolic blood pressure (mmHg)**	133.2 ± 14.7	139.1 ± 13.1	126.8 ± 13.8	<0.001	−13.28	−11.38
**Diastolic blood pressure (mmHg)**	77.7 ± 9.6	80.9 ± 9.8	74.1 ± 7.9	<0.001	−7.39	−6.11
**Waist circumference (cm)**	101.1 ± 13.2	106.4 ± 11.6	95.3 ± 12.4	<0.001	−11.95	−10.25
**Body mass index (kg/m²)**	29.3 ± 5.1	31.1 ± 4.8	27.3 ± 4.4	<0.001	−4.18	−3.53
**Atherogenic index (AI)**	3.8 ± 1.05	4.08 ± 1.15	3.51 ± 0.84	<0.001	−0.64	−0.50
**Visceral adiposity index (VAI)**	4.5 ± 3.3	5.8 ± 3.8	3.06 ± 1.7	<0.001	−2.94	−2.51

**Legend:** Values are expressed as mean ± standard deviation (SD). Comparisons between groups (MetS yes/no) were performed using the Student’s *t*-test for independent samples. CI = confidence interval. Statistically significant differences (p < 0.05) are shown in bold.

**Abbreviations:** MetS, metabolic syndrome; HDL, high-density lipoprotein cholesterol; LDL, low-density lipoprotein cholesterol; HbA1c, glycated hemoglobin; GFR, glomerular filtration rate; BMI, body mass index; WC, waist circumference; AI, atherogenic index; VAI, visceral adiposity index.

**Fig 2:**
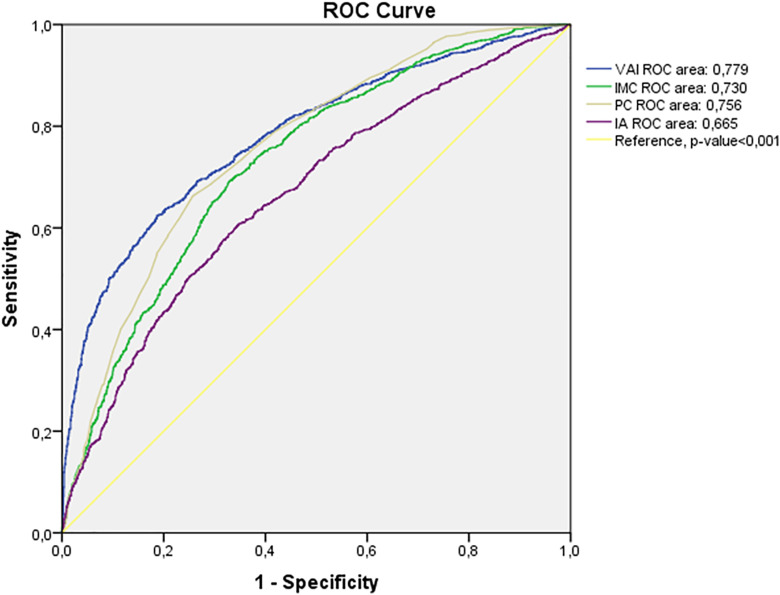
Receiver operating characteristic (ROC) curves for the dour diagnostic indices in relation to metabolic syndrome (MetS).

Receiver operating characteristic (ROC) curves were constructed for the four diagnostic indices—Visceral Adiposity Index (VAI), Body Mass Index (BMI), Waist Circumference (WC), and Atherogenic Index (AI)—to evaluate their performance in identifying metabolic syndrome (MetS). The area under the curve (AUC) was 0.779 (95% CI: 0.762–0.796) for VAI, 0.730 (95% CI: 0.711–0.749) for BMI, 0.756 (95% CI: 0.738–0.774) for WC, and 0.665 (95% CI: 0.642–0.688) for AI (Fig 2).

The Visceral Adiposity Index (VAI) showed the largest area under the curve (AUC = 0.779), followed by waist circumference (WC, AUC = 0.756), body mass index (BMI, AUC = 0.730), and atherogenic index (AI, AUC = 0.665); all comparisons were statistically significant (p < 0.001). The diagonal line represents the reference (AUC = 0.5).

The results of the four indices are summarized in Table 3. The area under the curve (AUC) values were 0.779 for the Visceral Adiposity Index (VAI), 0.756 for Waist Circumference (WC), 0.730 for Body Mass Index (BMI), and 0.665 for the Atherogenic Index (AI), all statistically significant (p < 0.001).

**Table 3:** Area under the ROC curve (AUC) for the four diagnostic indices.

Index	AUC	Asymptotic significance (p)	95% CI (Lower)	95% CI (Upper)
**Visceral Adiposity Index (VAI)**	**0.779**	<0.001	0.763	0.795
**Waist Circumference (WC)**	**0.756**	<0.001	0.739	0.773
**Body Mass Index (BMI)**	**0.730**	<0.001	0.712	0.747
**Atherogenic Index (AI)**	**0.665**	<0.001	0.646	0.684

AUC = area under the curve; VAI = visceral adiposity index; WC = waist circumference; BMI = body mass index; AI = atherogenic index.

All AUC values were statistically significant (p < 0.001), indicating that each index significantly discriminates the presence of Metabolic Syndrome (MetS). Among them, the VAI showed the highest diagnostic accuracy.

The optimal cut-off point for VAI was determined at 4.03 using the Youden index, yielding a sensitivity of 63%, specificity of 79%, positive predictive value (PPV) of 77%, and negative predictive value (NPV) of 66% (Table 4). The correlation coefficients between the four indices are shown in Table 5.

**Table 4:** Diagnostic performance of the Visceral Adiposity Index (VAI).

	MetS: Yes	MetS: No	Total
VAI positive	1032	302	1334
VAI negative	592	1181	1773
Total	1624	1483	3107

**Diagnostic parameters:** Sensitivity = **0.63**; Specificity = **0.79**; Positive Predictive Value (PPV) = **0.77**; Negative Predictive Value (NPV) = **0.66.**

MetS = Metabolic Syndrome; VAI = Visceral Adiposity Index.

**Table 5:** Pearson’s correlation coefficients between the four indices.

Index	AI	VAI	BMI	WC
AI	1	0.546**	0.181**	0.167**
VAI	0.546**	1	0.220**	0.265**
BMI	0.181**	0.220**	1	0.757**
WC	0.167**	0.265**	0.757**	1

*p* < 0.05 (*), *p* < 0.01 (**).

AI = Atherogenic Index; VAI = Visceral Adiposity Index; BMI = Body Mass Index; WC = Waist Circumference.

All correlations were positive and statistically significant, with the strongest association observed between BMI and WC (*r* = 0.757, *p* < 0.001). The VAI showed moderate correlations with both AI and anthropometric indices, reflecting its composite nature that integrates lipid and adiposity parameters.

## Discussion

As a real and growing public health problem, metabolic syndrome (MetS) must be detected and prevented early using the most appropriate and feasible tools in clinical practice. In this research study, conducted in a primary care setting in the Spanish population of Aragón (Northern Spain), the value of the Visceral Adiposity Index (VAI) as a predictor of MetS was evaluated and compared with three other indices (atherogenic index, waist circumference, and body mass index), which are the most commonly used in our environment. The characteristics of MetS were also analyzed in the same population, selected according to the availability of the variables required for the calculation of these indices.

Our sex- and age-specific patterns broadly mirror findings from large European datasets, but with noteworthy differences. In the DARIOS collaborative study in Spain, MetS prevalence was higher in men up to midlife, converged in the 55–64 range, and became higher in women thereafter—consistent with our age-stratified results [25]. However, the magnitude of sex differences in our cohort was slightly larger in the < 45 and 45–65 groups. This may reflect differences in population structure (primary-care users with chronic conditions in our study vs. mixed community samples in DARIOS), temporal trends in obesity and diabetes, and regional variation in lipid-lowering therapy uptake. Likewise, results from SIDIAP in Catalonia reported high co-occurrence of hypertension and obesity among MetS cases; we observed a similar pattern, although diabetes was proportionally more frequent in our cohort [26]. Differences in case mix (older age and higher chronic disease burden in CRONAP), clinical coding practices, and laboratory testing density could contribute to these discrepancies. Overall, the convergence across Spanish/European cohorts supports external consistency, while between-study differences highlight the influence of setting, time period, and treatment patterns on MetS components.

All analytical variables were higher in the MetS group, except HDL-cholesterol, which was logically lower. Significant differences were found between groups in all measures except total and LDL-cholesterol, contrary to most published evidence. This unexpected result may be related to adherence to the Mediterranean diet, higher physical activity, or statin therapy in our region, but further research is required to clarify these factors.

Regarding comorbidities, our data confirmed significant associations between MetS and diabetes (72.3%), obesity (69.3%), and hypertension (61.4%). Similar relationships have been reported in the SIDIAP database in Catalonia, where hypertension (86.5%) and obesity (56.6%) were the most frequent comorbidities [26]. In line with our findings, other studies have shown that fasting glucose, HbA1c, and triglyceride levels are significantly higher in individuals with MetS, supporting their key role in the pathophysiological definition of the syndrome.

Our results also corroborate the evidence that anthropometric indices such as BMI, WC, and especially VAI are elevated in the presence of MetS. In a study in working adults, the VAI achieved an AUC = 0.85, with sensitivity = 74.7% and specificity = 85.5%, slightly higher than in our study, possibly due to a younger, male-dominant sample [22]. In a large multi-ethnic population in rural China (Uyghur, Kazakh, and Han), the VAI correlated significantly with MetS irrespective of ethnicity, age, or other covariates (AUC = 0.789) [27]. A meta-analysis of 18 studies further confirmed a moderate-to-high diagnostic accuracy of VAI for predicting MetS (pooled AUC = 0.847; sensitivity = 78%; specificity = 79%) [28]. Variability among studies was mainly attributed to differences in diagnostic criteria, ethnicity, and lifestyle, but the threshold effect was not significant, suggesting that different VAI cut-off points did not materially affect diagnostic performance.

The heterogeneity of VAI cut-off points reported across studies likely reflects a combination of ethnic background, age distribution, sex composition, clinical setting, and underlying lipid profiles. For example, Mediterranean cohorts often show higher HDL and favorable triglyceride distributions than non-Mediterranean groups, potentially shifting optimal thresholds. Age is particularly relevant; adipose tissue distribution and lipid metabolism change with aging, and Amato et al. reported age-stratified VAI thresholds with materially different optima across decades of life [17]. Sex differences also matter, because the VAI formula is sex-specific and the relative contribution of WC, TG, and HDL varies by sex across populations. Finally, treatment exposure (e.g., statins, antihypertensives) and selection of clinical vs. community samples can alter lipid levels and anthropometry at baseline, thereby influencing ROC-derived cut-offs. These factors argue against a single universal threshold and support context-specific calibration (e.g., by age/sex bands) when VAI is applied for screening in European primary care.

The current findings are consistent with earlier work in Mediterranean populations. Gil Llinás et al. (2017) reported that the VAI, the lipid accumulation product (LAP), and the triglyceride-to-HDL ratio were the most accurate indices for predicting MetS using NCEP ATP III criteria, while body roundness index (BRI) and waist-to-height ratio performed better under IDF definitions [29]. In Brazilian and Asian cohorts, Roriz et al. (2014), Oh et al. (2018), and Liu et al. (2024) demonstrated similar superiority of VAI-based models over single anthropometric measures, and proposed population-specific adjustments (e.g., NVAI, AVAI) incorporating age or blood pressure to improve discrimination [15,30,31]. Although some studies, such as the Baependi Heart Study, found stronger associations of BMI and WC with hypertension than with VAI, differences in outcomes (hypertension versus MetS) and ethnic composition may explain these discrepancies [30]. Overall, these results reinforce the relevance of indices that combine anthropometric and biochemical data, which are less influenced by ethnicity and more directly linked to visceral fat dysfunction.

In this context, the VAI appears as a comprehensive and accessible indicator for assessing cardiometabolic risk in Mediterranean populations. Its superior discriminative performance compared with BMI, WC, and AI in our study supports its clinical usefulness. Unlike BMI or WC alone, the VAI reflects both adiposity and lipid metabolism, providing a multidimensional view of metabolic health that can be applied in primary care without additional cost. Given its reliance on routinely collected data, it represents a feasible, evidence-based tool for nursing and community health professionals to stratify metabolic risk and guide preventive interventions.

This study has several strengths, including the large sample size from a real-world primary care database, standardized data capture across a single public health system, and the joint evaluation of anthropometric and biochemical indices using established criteria and robust statistical methods.

Important limitations should be acknowledged. First, the retrospective, cross-sectional design precludes causal inference regarding the relationships between indices and MetS. Second, residual confounding is possible: medication use (e.g., lipid-lowering, antihypertensive, and glucose-lowering drugs), dietary patterns, smoking intensity, and physical activity were not fully captured, and could partly account for the lower LDL-cholesterol and total cholesterol observed in the MetS group. Third, although we included all CRONAP patients with complete data for VAI calculation at the prespecified baseline, selection bias cannot be ruled out because individuals without complete anthropometric/biochemical data were excluded; these individuals may differ systematically from those included (e.g., healthier users with fewer labs or, conversely, sicker users with incomplete records). Fourth, misclassification of MetS components is possible due to reliance on routine care data and a single time-window for defining variables; repeated measurements might provide more stable estimates. Fifth, we used NCEP ATP III criteria; while widely adopted, these may not align perfectly with IDF or WHO definitions, limiting direct comparability with studies using alternative criteria. Finally, our findings pertain to a Mediterranean, largely European population within a universal healthcare system; generalizability to non-European or non-Mediterranean contexts should be evaluated cautiously and ideally validated in independent European cohorts.

Although selection bias cannot be excluded, we minimized it by prespecifying a baseline date (six months before the COVID-19 lockdown) and including all CRONAP patients with the complete variables required to compute VAI; nevertheless, excluding individuals with incomplete data may have introduced differences between included and excluded patients. The full patient-selection pathway, including inclusion/exclusion criteria and counts at each step, is shown in Fig 1 (flowchart).

Taken together with Spanish multicenter data (e.g., DARIOS) and regional primary care evidence (e.g., SIDIAP), our results support the applicability of VAI-based screening in European Mediterranean settings. Nevertheless, given the variability in lipid profiles, statin exposure, and adiposity distribution across Europe, local calibration and external validation are advisable—ideally with age- and sex-specific thresholds—to ensure optimal performance when deploying VAI in routine European primary care.

## Conclusions

Metabolic syndrome (MetS) represents a growing global health challenge due to its close association with cardiometabolic risk. This increasing burden highlights the need for reliable, cost-effective, and easily applicable tools for early detection. In this regard, the Visceral Adiposity Index (VAI) emerges as a practical, low-cost, and accessible screening strategy with good discriminative performance.

In our study, all four evaluated anthropometric and biochemical indices— Visceral Adiposity Index – VAI, Body Mass Index – BMI, waist circumference – WC, and atherogenic index – AI, showed significant associations with MetS, confirming their utility as predictors of metabolic alterations. Among them, the VAI demonstrated superior discriminatory capacity, particularly for ruling out false positives, and exhibited a closer relationship with the atherogenic index, likely due to the shared lipid components in their calculation formulas. The cut-off point derived in our population was higher than in other studies, yielding the best balance between sensitivity and specificity for our Mediterranean cohort.

The characteristics of our population, including the inversion of MetS prevalence by sex and age and its strong association with diabetes, obesity, and hypertension, are consistent with findings from other European studies, supporting the extrapolation of these results to broader Mediterranean and European populations. The predominance of analytical and clinical alterations (elevated triglycerides, glucose, HbA1c, BMI, and blood pressure) further reinforces the clinical relevance of early metabolic screening in primary care.

However, given the retrospective and cross-sectional design, caution is warranted in interpreting these findings. Prospective, multicenter studies are needed to confirm the predictive performance of the VAI and to establish standardized cut-off points by sex and age for European populations.

From a clinical perspective, the VAI could be readily integrated into primary care and nursing workflows, since it requires only routinely collected parameters already available in most electronic health records. Incorporating VAI-based assessment into preventive health programs could facilitate early identification of individuals at metabolic risk, allowing timely lifestyle or pharmacological interventions and improved follow-up of cardiometabolic health.
