# Correction: Comparison of four diagnostic indices for metabolic syndrome in a Northern Spanish population

**DOI:** 10.1371/journal.pone.0345124

**Published:** 2026-03-16

**Authors:** Liliana Bilbie Lupchian, Bárbara Oliván-Blázquez, Edgar Peña-Galo, Marta Domínguez-García, María Antonia Sánchez-Calavera

There are errors in the Funding statement. The correct Funding statement is as follows: This work was funded by the Carlos III Health Institute (ISCIII), with funds from the Research Network on Chronicity, Primary Care and Health Promotion (RICAPPS, RD24/0005/0004), which is part of the Cooperative Research Networks Oriented to Health Outcomes (RICORS) (Carlos III Health Institute), co-funded by the European Union.
